# Does Perceived Stress of University Students Affected by Preferences for Movie Genres? an Exploratory Cross-Sectional Study in China

**DOI:** 10.3389/fpsyg.2021.761340

**Published:** 2021-11-26

**Authors:** Ning Qiao

**Affiliations:** School of Arts, Peking University, Beijing, China

**Keywords:** perceived stress, mental health, preferences for movie genres, university students, psychology

## Abstract

This study aims to explore whether different preferences for movie genres were related to different perceived stress of college students. An online questionnaire was designed and it was filled out by 1,549 students voluntarily. The 10-item perceived stress scale (PSS-10) and multinomial logistic regression were used to access the perceived stress and the association between the movie preference genres. Over 90% of participants had mild to serious levels of stress. Differences were found between participants with different stress perception states in terms of smoking history, active exercise, and sleep duration (*p* < 0.05). The participants who showed a preference for suspense movies more probably had lower stress [relative risk ratio (RRR)1 = 0.34, RRR2 = 0.26, *p* < 0.05], while students who showed preferences for crime film and disaster film more probably had higher stress (RRR = 2.03, *p* < 0.05, RRR = 3.15, *p* < 0.05). And the significant gender gap in different film genre preferences was observed in this study (*p* < 0.05). The males who showed preference for horror movies were more probably to have moderate stress (*OR* = 3.68, *p* < 0.05), and females who showed a preference for disaster movies were more probably to have high stress (*OR* = 3.27, *p* < 0.05). The perceived stress of Chinese university students is high after 1.5 years of coronavirus disease (COVID-19) pandemic. The personal preferences for certain film genres were significantly associated with perceived stress. As different film genre preferences, such as the preference for disaster, crime, and horror, are associated with high perceived stress, it may turn out to be useful to pay more attention to an individual’s film viewing. The teachers need to be concerned with the media usage history and preferences of their students and may advise students with high-level stress to avoid potentially harmful media content.

## Introduction

Stress is a state or feeling that people experience when they perceive that the demands placed on them surpass their personal and coping capabilities. The acute and chronic stress have been proved to dysregulate the hypothalamic-pituitary-adrenal (HPA) axis and the sympathetic nervous system (SNS) and lead to vulnerability to psychiatric complications (Kudielka and Kirschbaum, 2005; Carver, 2007). Stress commonly exists among college students, as they frequently encounter it, possibly caused by experiences of academic failure, high expectations from parents, and changes in friendships under this unique developmental period of transition from adolescence into young adulthood (Borjalilu et al., 2015). Several studies also revealed that prolonged periods of high levels of stress strongly affect mental health and also have unfavorable effects on academic performance (Dyrbye et al., 2006; Regier et al., 2013; Saleh et al., 2017; Rogowska et al., 2020).

Stress among higher institution students has greatly increased over the past few decades, with the studies showing that nearly 40–60% of qualified nurses and first-year medical students in China have mild to severe stress (Cheung et al., 2016; Khalid et al., 2019). At the end of 2019, the coronavirus disease (COVID-19) outbreak, the epidemiological data suggested that most college students suffered moderate to high stress during the COVID-19 pandemic (Hathaway et al., 2021; Kostic et al., 2021; Li Z. et al., 2021; Lippke et al., 2021; Marcen-Roman et al., 2021; Okoro et al., 2021). A study of college students from the United States indicated that 60.8% of them faced increases in anxiety, 54.1% faced loneliness, and 59.8% faced depression (Lee et al., 2021). And a study carried out in China examined the mental health of 89,588 college students, of which 41.1% of them reported anxiety symptoms (Ma et al., 2020). Multiple stressors in pandemic, such as the fear and worry about the health of their own and their loved ones, difficulty in concentrating, decreased social interactions due to physical distancing, and increased concerns on academic that increased vulnerability to psychological health problems of college students, led to the high perceived stress among college students (Li Y. et al., 2021).

Film viewing is a popular leisure activity that people spend more time watching movies than on any other activity (Sinyor et al., 2020). In recent years, movies have not only been a source of endless entertainment but have a potential educational and therapeutic impact as well (Murphy et al., 2015; Chiong-Rivero et al., 2021). Cultivation theory (Gerbner et al., 1986) suggests that movies can subtly influence peoples’ perception of reality, such as cumulative exposure to sad, and disturbing film content has the potential to increase or maintain tension among viewers, while happy, jolly media contents could help to maintain a positive emotional state. Recipients may strive for more positive and pleasurable moods or seek to maintain their current emotional state. Mangala and Thara (2009) found that cinema can be used very effectively to improve awareness about mental health issues. Furthermore, viewing humorous movies can help to reduce levels of psychopathology, anger, anxiety, and depression symptoms and an improvement in society (Gelkopf et al., 2006). And horror movies might help the audience to relieve the pressure of life and obtain psychological pleasure (Nan, 2014). Furthermore, people who engaged more frequently with horror movies showed greater positive resilience during the COVID-19 pandemic (Scrivner et al., 2021). The film could be tailored to the social norms and values of a specific population, which supports the use of film as a future intervention to address health barriers and the perception of stress to boost mental health (Hoang et al., 2021).

A variety of psychological concerns have been shown in movies for over a century through fascinating plots and characters. Studies have pointed out that there is a heterogeneous network approach to predict individuals’ mental health based on personal preferences (Liu et al., 2021). And the associations between film preferences and risk factors for suicide have been found (Till et al., 2014). Different film types involve different types of scenes, such as comedy movies involving funny scenes, suspense movies involving suspenseful scenes, horror movies may involving violent scenes, which have powerful effects on audiences, and they effectively guide psychological processes, stress perceptions, and create experiences that evolve over time (Schmälzle and Grall, 2020). The preference of movie genres could roughly assess the individual’s viewing habits and possible exposure on the screen (Hawkins et al., 2001). In the long term, the need for affect (NFA) may mediate film preference; if the basic NFA was not satisfied, the individuals’ perceptions of pressure might increase (Martin, 2019). As a result, an increased likelihood of mental health problems, such as depression, has been hypothesized when individuals perceive stress as uncontrollable, unpredictable, and severe and believe that the resources could cope with it are insufficient (Hammen, 2005).

The perception of uncontrolled stressors has been found to have profound physiological, cognitive, and motivational consequences and increase susceptibility to emotional disturbances (Maier and Watkins, 2005). We assume that long-term preference for movies genres may reduce or increase students’ perception of stress. Researches about associations of personal film preferences with perceived stress were relatively scarce. This study aims to explore the association between the different preferences for movie genres and the risk for perceived stress of Chinese university students, which look forward to providing a theoretical basis on preferences for movie genres as the predictor of perceived stress for the future and as an intervention tool.

## Materials and Methods

### Participants and Study Design

The study was anonymous and does not involve personal privacy issues, and the detailed description of the study and the tips about the questionnaire filling were shown on the questionnaire completion website. A web-based survey (a closed investigation) was carried out, using convenience sampling in a university. Based on the single population proportion formula (Daniel, 1999), n=(Z⁢α2⁢ 2M⁢O⁢E2*p⁢(1-p)). We set the α = 0.05, $Z\,\frac{\alpha }{2}$ = 1.96, MOE = 0.05, and *p* = 50%, the calculated *n* = 383. The population was the university students who, following the notifications that were put on social media platforms such as Wechat and Tencent QQ, had agreed to participate in online surveys voluntarily. The survey period lasted for 4 weeks to enlist more participants to participate in. We recruited 1,788 university students in Beijing, China, including undergraduates and graduate students. Volunteer students filled out the questionnaire by a web link on the social media in July 2021, which time was after the examination that could reduce the participants’ concerns that could keep the assurance of data reliability. The exclusion criteria for this survey data are as follows :(1) the questionnaire data with logical errors, (2) the questionnaire data with inconsistent reconfirmation questions such as gender and age, and (3) participants whose amount of movie watching is <1 year and more than three movies a day. A total of 1,549 questionnaires were collected after excluding invalid questionnaires. The majors of participants include two categories: natural sciences (46%) and social sciences (54%). Natural science majors include medical, agricultural, and science majors, while art, management, and education majors are social sciences.

### Measurement

#### Questionnaire

The questionnaire was self-administered and specifically designed for the target population, with a presurvey being conducted 2 months before the formal survey. The final version questionnaire was completed with a pilot study in which 85 students participated in the pertest by the one-faced-one electronic questionnaire link and repeated discussions with experts. The reliability of Cronbach’s alpha coefficient of the questionnaire was 0.82. The Kaiser-Meyer-Olkin (KMO) validity statistical test (KMO = 0.79) and Bartlett sphere 21 city test (*p* < 0.0001) were used. The questionnaire contained both open-and close-ended questions that were grouped into three sections: (1) the informed consent detailed information about this study (2) sociodemographic characteristics and lifestyle of university students, and (3) the preference for movie genres and perceived stress scale.

#### Perceived Stress Scale

The 10-item perceived stress scale (Cohen et al., 1983) comprised 10 items: six positive items and four negative items rated on a five-point Likert scale (0 = Not at all/Never,1 = Rarely, 2 = Sometimes, 3 = Fairly often, and 4 = Very often). There is a norm table for the PSS-10 item inventory (e.g., “In the last month, how often have you been upset because of something that happened unexpectedly?”). This study used the Chinese version PSS to measure the extent of self-aware stress and the belief of university students to perceive their life over the past 30 days before the survey. This PSS has shown that high internal consistency reliability, as measured by Cronbach’s α, was 0.85 and the test-retest reliability coefficient was 0.7 (Lu et al., 2017; Huang et al., 2020). All items were added; the higher the score, the greater the self-perceived stress. The total score ranged from 0 to 40. And a score ranging from 0 to 13 would be considered low stress; a score between 14 and 26 represents moderate stress, and a score ranging from 27 t0 40 represents a high level of perceived stress.

#### Genres of Movie Preference

The preference of movies was set as the primary predictor based on eight movie genres, referring to the genres of the standard to the previous research (Scrivner et al., 2021). The participants were asked to what extent they agreed (five-point scales, dislike to strongly like) with each of eight statements that said, “I think I like the ______ movies.” The eight genres of movies were horror (including the zombie, psychological thriller, and supernatural), disaster, science fiction (including superheroes and alien-invasion), suspense, crime, action, comedy, and romance. The participants were also asked to rate the extent to which they agreed (five-point scales, strongly disagree to strongly agree) with a statement of generic enjoyment of video entertainment, “I enjoy watching movies.”

### Confounding Variables

The basic socioeconomic status variables and lifestyle variables were employed as confounding variables. The basic socioeconomic variables include family information and self-information. The self-information included gender, age, major, learning grade, region, the living place, and history of the disease (the disease including hypertension diabetes, asthma, cardiovascular disease, and a history of surgical procedures). Whether the only child or not, the expenses beyond food expenses, education level of parents, and family structure were called the family information. Lifestyle variables included the history of smoking, which was defined as whether or not the participants had smoked more than one cigarette in the past year, history of drinking was defined as whether or not the participants had intake any alcoholic beverage of 200 ml or more in the past year, and active exercise, which was defined as the initiative to do physical activities other than school physical education classes over the past 30 days.

### Statistical Analysis

All analyses were conducted using Stata version 15.0 (Stata Corporation, College Station, TX, United States). Deviations from normality and univariate outliers were screened for all variables. Descriptive statistics were used to characterize the three stress level participants in terms of basic socioeconomic status variables and lifestyle variables. The chi-square test was used for categorical variables and ANOVA for continuous variables based on the normally distributed data. The preferences for the eight movie categories were recoded as “neutral,” “dislike,” and “like.” The multinomial logistic regression was used to assess the sociodemographic determinants of preference for movie genres, and the multilevel logistic regression was presented to assess the association between movie preference and perceived press. The following three models were used. Model 1 was adjusted for preference for different genres of movies, Model 2 was further adjusted for basic socioeconomic status variables, and Model 3 was further adjusted for lifestyle variables. The results are reported for odds ratio (OR), relative risk ratio (RRR), and 95% CI.

## Results

Table 1 demonstrates that there were only 94 participates in the low press and most participants (1390) were in the moderated press. The mean age of participants was 20.69 years (SD 1.90) and nearly 50% of participants’ expenses other than food expenses were more than 600 yuan. The following data were obtained: 64.82% were females, 54.68% were from a rural area, and 80.76% lived in a dormitory during the study period. Two-thirds of participants did not have a romantic relationship. The participants reported that the mean hours of sleep were 7.75 (SD 1.30). Besides, differences were found between participants with different stress perception states in terms of smoking history, active exercise, and sleep duration (*p* < 0.05).

**TABLE 1:** Characteristics of participants.

		**Low stress**	**Moderate stress**	**High stress**	**Total**	***P*-value**
		***N* = 94**	***N* = 1,390**	***N* = 65**	***N* = 1,549**	
Age		20.31 ± 1.57	20.69 ± 1.91	21.05 ± 2.01	20.69 ± 1.90	0.047*
Gender	Male	34 (36.17%)	486 (34.96%)	25 (38.46%)	545 (35.18%)	0.83
	Female	60 (63.83%)	904 (65.04%)	40 (61.54%)	1,004 (64.82%)	
Learning stage	BS1∼2	73 (77.66%)	947 (68.13%)	34 (52.31%)	1,054 (68.04%)	0.019*
	BS3–4/5	14 (14.89%)	314 (22.59%)	23 (35.38%)	351 (22.66%)	
	MS	7 (7.45%)	129 (9.28%)	8 (12.31%)	144 (9.30%)	
One child family	Yes	43 (45.74%)	607 (43.67%)	30 (46.15%)	680 (43.90%)	0.86
	No	51 (54.26%)	783 (56.33%)	35 (53.85%)	869 (56.10%)	
Education level of father	Low	40 (42.55%)	619 (44.53%)	27 (41.54%)	686 (44.29%)	0.32
	Medium	37 (39.36%)	450 (32.37%)	18 (27.69%)	505 (32.60%)	
	High	17 (18.09%)	321 (23.09%)	20 (30.77%)	358 (23.11%)	
Education level of Mather	Low	48 (51.06%)	716 (51.51%)	30 (46.15%)	794 (51.26%)	0.35
	Medium	35 (37.23%)	419 (30.14%)	21 (32.31%)	475 (30.66%)	
	High	11 (11.70%)	255 (18.35%)	14 (21.54%)	280 (18.08%)	
Single parent family	Yes	9 (9.58%)	111 (7.99%)	5 (7.70%)	125 (8.07%)	0.19
	No	85 (90.43%)	1,279 (92.01%)	60 (92.31%)	1,424 (91.93%)	
Area	Urban	35 (37.23%)	629 (45.25%)	38 (58.46%)	702 (45.32%)	0.030*
	Rural	59 (62.77%)	761 (54.75%)	27 (41.54%)	847 (54.68%)	
Expenses other than food expenses	< 600¥	41 (43.62%)	732 (52.66%)	28 (43.08%)	801 (51.71%)	0.017*
	600–900¥	28 (29.79%)	350 (25.18%)	12 (18.46%)	390 (25.18%)	
	>900¥	25 (26.60%)	308 (22.16%)	25 (38.46%)	358 (23.11%)	
Living place	Dormitory	88 (93.62%)	1,117 (80.36)	46 (70.77%)	1,251 (80.76%)	< 0.001*
	Others	6 (6.38%)	273 (19.64%)	19 (29.23%)	298 (19.24%)	
Romantic relationship	Yes	26 (27.66%)	517 (37.19%)	17 (26.15%)	560 (36.15%)	0.041*
	No	68 (72.34%)	873 (62.81%)	48 (73.85%)	989 (63.85%)	
History of disease	No	93 (98.94%)	1,330 (95.68%)	57 (87.69%)	1,480 (95.55%)	0.002*
	Yes	1 (1.06%)	60 (4.32%)	8 (12.31%)	69 (4.45%)	
History of Smoking	No	80 (85.11%)	1,045 (75.18%)	43 (66.15%)	1,168 (75.40%)	0.020*
	Yes	14 (14.89%)	345 (24.82%)	22 (33.85%)	381 (24.60%)	
History of drinking	No	25 (26.60%)	355 (25.54%)	12 (18.46%)	392 (25.31%)	0.42
	Yes	69 (73.40%)	1,035 (74.46%)	53 (81.54%)	1,157 (74.69%)	
Active exercise	Yes	72 (76.60%)	899 (64.68%)	37 (56.92%)	1,008 (65.07%)	0.024*
	No	22 (23.40%)	491 (35.32%)	28 (43.08%)	541 (34.93%)	

*p < 0.05. Year of study: BS1–2: the first and second grade of bachelor study, S3–4/5: the third to the fourth or fifth grade study, MS: all grades of the master study.

After the adjustment for covariates, Table 2 showed the year of study, only child family, educational level of parents, and expenses other than food expenses had no significant influence on participants’ film genres preference (*p* > 0.05). The age only affected participants’ preference for romance movies, with the older they got, the more they liked romance movies (*OR* = 1.14, *p* < 0.05). And participants without a romantic relationship showed less preference for romance films (*OR* = 0.74, *p* < 0.05). A significant gender gap in different film genre preferences was observed in our study. The female participants showed less preference for horror, disaster, science fiction, and crime action and showed more preference for romance movies than male participants (*p* < 0.05). The participants who lived in the dormitory showed less preference for horror, disaster, and suspense, while more preference for comedy than those who lived in other places (*p* < 0.05).

**TABLE 2:** Odds ratio (OR) estimated by logistic regression on associations between eight film genres preferences and sociodemographic factors.

**Variables**	**Subgroup**	**FP for horror**	**FP for disaster**	**FP for science fiction**	**FP for suspense**	**FP for crime**	**FP for action**	**FP for comedy**	**FP for romance**
Age		1.05	1.05	0.98	1.03	1.06	1.06	1.02	1.14*
Gender	Female	0.66*	0.53*	0.50*	0.86	0.70*	0.40*	1.21	1.38*
Year of study	3rd-4th/5th	0.78	0.98	1.37	1.14	1.01	0.97	1.17	0.83
	Postgraduate	0.92	0.81	1.39	1.17	1.13	1.15	1.08	0.56
Area	Rural	1.01	0.83	0.95	0.80	0.70*	0.91	1.01	1.11
One child family	No	0.87	0.98	0.98	0.88	0.85	1.21	1.30	1.22
Education level of father	Medium	1.08	1.09	0.96	1.03	0.93	0.88	1.01	1.00
	High	1.33	1.33	1.26	1.29	1.34	0.87	1.08	0.91
Education level of mother	Medium	1.25	1.28	1.07	1.25	1.15	0.98	0.91	0.92
	High	0.92	1.01	1.20	0.90	1.05	0.96	0.73	0.93
Family structure	Steam family	0.89	0.83	1.16	1.14	0.94	0.98	1.86*	1.22
	Others	1.09	0.40	0.39	0.60	0.26*	0.65	0.82	0.73
Expenses other than food expenses	600–900¥	0.95	0.95	0.98	1.01	1.19	1.30	1.24	1.10
	>900¥	0.82	0.96	0.94	0.83	1.18	1.29	1.33	1.19
Living place	Others	3.04*	2.39*	1.16	1.71*	1.07	1.20	0.49*	1.21
Romantic relationship	No	0.84	0.94	1.10	0.97	0.90	0.91	0.96	0.74*
History of disease	Yes	1.92*	2.27*	0.97	1.10	1.15	0.78	0.97	0.95

*p < 0.05. FP, Film Preference. The dislike for film genres was treated as the reference category in the film genres preference options. And the male, the 1st–2nd year of study, the only child, the low education of parents, the single-parent family, expenses other than food expenses lower than 600¥, living in the dormitory, having a romantic relationship, and having a history of disease were treated as the reference categories.

Table 3 shows the association between press level and different film genre preferences in the fully adjusted model. The male participant who showed the preference for horror movies might be more probably at the moderate-press level (RRR = 3.68, *p* < 0.05). And participants who showed a preference for disaster movies were more probably at the high-press level (RRR = 3.15, *p* < 0.05), with the same significant difference in females were also found (RRR = 3.27, *p* < 0.05). The participants who showed a preference for suspense movies more probably have the lower press level (RRR = 0.34, RRR = 0.26, *p* < 0.05). And the difference in moderate-press level of male participants was more significant than female participants (RRR _male_ = 0.12, RRR _female__=_ 0.26). Additionally, we found that the students who showed a preference for crime films were more probably at a higher press level (RRR = 2.03, *p* < 0.05).

**TABLE 3:** Relative risk ratios (RRR) estimated by multinomial regression on associations between pressed stress and film genres preferences.

**Film genres preferences**	**Moderate-press (RRR 95%CI)**			**High press (RRR 95%CI)**		
	**All**	**Female**	**Male**	**All**	**Female**	**Male**
FP for horror	1.39 (0.79, 2.44)	0.84 (0.42, 1.66)	3.68* (1.36, 9.98)	0.91 (0.38, 2.18)	0.72 (0.23, 2.21)	1.47 (0.35, 6.19)
FP for disaster	1.71 (0.96, 3.06)	1.79 (0.90, 3.54)	1.64 (0.54, 5.02)	3.15* (1.28, 7.78)	3.27* (1.09, 9.86)	3.52 (0.66, 18.73)
FP for science fiction	0.81 (0.42, 1.54)	0.72 (0.35, 1.51)	1.02 (0.25, 4.12)	1.10 (0.39, 3.10)	1.34 (0.40,4.53)	0.54 (0.07, 4.30)
FP for suspense	0.34* (0.17, 0.66)	0.50 (0.22, 1.13)	0.14* (0.04, 0.50)	0.26* (0.09, 0.73)	0.26* (0.07, 0.96)	0.24 (0.04, 1.52)
FP for crime	2.03* (1.08, 3.82)	1.81 (0.84, 3.90)	2.48 (0.73, 8.49)	2.23 (0.73, 6.80)	2.2 (0.61, 8.50)	2.83 (0.34, 23.58)
FP for action	1.13 (0.60, 2.12)	1.22 (0.60, 2.50)	0.86 (0.19, 3.81)	1.00 (0.36,2.80)	0.78 (0.24, 2.50)	1.58 (0.15, 16.71)
FP for comedy	0.61 (0.22, 1.72	0.83 (0.21, 3.25)	0.37 (0.06, 2.31)	0.49 (0.12, 2.05)	0.46 (0.07, 2.89)	0.37 (0.03, 4.55)
FP for romance	1.26 (0.71, 2.23)	0.95 (0.44, 2.05)	1.53 (0.59,3.97)	0.98 (0.41, 2.34)	1.10 (0.34, 3.64)	0.70 (0.17, 2.86)

*p < 0.05. FP, Film Preference. The dislike for film genres was treated as the reference category in the film genres preference options, and the low stress was treated as the reference category in pressed stress. The model was adjusted for different preferences for film genres, gender, age, learning grade, region, living place, disease status, family structure, the history of smoking, history of drinking, and active exercise.

## Discussion

Excessive perceived stress is associated with negative physical health outcomes and exacerbation of mental health symptoms. The nervous system mobilizes the body in response to a threat by releasing some hormones (e.g., epinephrine, norepinephrine, glucocorticoids) and inhibiting others (e.g., insulin, estrogen, progesterone) (Carver, 2007). Nevertheless, the perceived stress commonly existed among college students of this study. Over 90% of the participants had relatively mild to severe perceived stress, which was significantly higher than the study conducted in the 2020 Chinese COVID-19 pandemic (Zhan et al., 2021), and higher than the Spanish students almost 1 year after the COVID-19 pandemic (Marcen-Roman et al., 2021). However, a higher proportion was reported in nursing students (96.01%) in Pune (Sheroun et al., 2020). This might closely related to the severity and isolation modes of COVID-19 prevention and control. And the survey time, locations and the characteristics of the investigation population would also lead the difference. With so many students under medium and high levels of perceived stress, how to effectively reduce stress is the more important thing. The film has been indicated to be a future intervention to address health barriers and the perception of stress to boost mental health (Hoang et al., 2021).

The preferences for movie genres can indicate both individuals’ movie viewing habits and possible exposure on the screen for a long term (Hawkins et al., 2001). A significant gender gap in different film genre preferences was observed in our study, which was consistent with previous studies (Duuren, 2008; Wuhr et al., 2017; Zhang et al., 2019). Similarly, female participants expressed more preference for romance movies than males and male participants expressed more preference for action movies, horror movies, crime movies, and science fiction movies in this study. As more opportunities for the participants with a romantic relationship to watch the romance film when dating, thus they might show the more preference for romance movies. Different film types involve different types of scenes, such as comedy movies involving funny scenes, suspense movies involving suspenseful scenes, horror movies may involve violent scenes, which have powerful effects on audiences, and they effectively guide psychological processes, stress perception, and create experiences that evolve over time (Schmälzle and Grall, 2020). These discrepancies may be one of the reasons for the sex differences in stress level observed for the same-film preference.

In this study, the male participants with a preference for horror movies were more probably at the high level of stress than the female participants who show a preference for horror movies. The underlying mechanism of this gender difference might involve differential actions of male and female sex hormones (Angold et al., 1998). The pattern of the response to the stressful stimulus in the activation of physiological pathways including the HPA axis and the autonomic nervous system is sex-specific (Kudielka and Kirschbaum, 2005; Kajantie and Phillips, 2006). The circulating ovarian hormones tend to offer neuroprotection against neurocognitive, affective, and HPA dysregulation. Additionally, men put less emphasis on the need to manage their stress than women do, which resulted from mood management-based processes (Matud, 2004). Previous studies have pointed out that watching horror movies does not cause real harm to the human body, but will secrete a kind of adrenaline to produce a pleasant enjoyment. Just like when playing skydiving and bungee jumping (Wuhr et al., 2017; Martin, 2019; Scrivner et al., 2021). The cerebral cortex, glands, and other development of university students are not complete, and the body is still in an important growth period. If they always watch horror movies, it will cause dysfunction, high perceived stress, and other problems. The psychological health will also be adverted. The results of the present study warrant more research on the link between perceived stress and the preference for horror movies, which also suggests that when using movies as psychological interventions in the future, gender differences should be addressed, and different types of movies should be selected according to the gender.

Nevertheless, individuals with preferences for suspense had significantly lower perceived stress in this study. This outcome is in line with the result of Nabi’s study (Nabi et al., 2016). The individuals with preferences for suspense movies more probably have lower stress due to the repeated exposure to suspenseful movies, which might have a lower skin conductance level (SCL) and ECG that result in affective habituation or desensitization to repeated stimuli (Chun et al., 2020). And extant research has found that suspenseful film clips led to decreases in cortisol (Hubert and de Jong-Meyer, 1991). The most central indicator is the hormone cortisol that a glucocorticoid produced within the adrenal gland, which is considered the “stress hormone” as it is produced in response to stress and anxiety. Stressed individuals produce greater amounts of cortisol, and if stressors persist, cortisol levels increase, which, over time, can increase blood glucose (associated with diabetes), increase high blood pressure, and interfere with immune system functioning (Carver, 2007; Nabi et al., 2016). The suspense movies might be used to do the materials for mental health intervention for college students with high levels of stress in the future, especially for female college students.

The preference for disaster movies among high perceived stress individuals found in the present study is particularly noteworthy. Although, the finding is not surprising, since these genres provide movies that typically display violence, widespread chaos that can occur during real world (Mrug et al., 2015; Fanti et al., 2017; Steiger et al., 2019). The information the participants who prefer watching these movies obtain vicariously from an imagined disaster apocalypse might lead to higher perceived stress in the real world (Clasen, 2019). It has been demonstrated the blood pressure of youth exposed to high levels of televised violence will rapidly increase (Mrug et al., 2015). However, some previous studies also indicated that the viewers are not able to identify the unrealistic settings typically portrayed in disaster movies. These scenes are the cognitive testing ground where lead cognitively and emotionally model the experience of living through the worst to reflect on a radical change in emotion. Indeed, it is quite likely that we may find that the media we find psychologically distracting and enjoyable (e.g., video games, humor) may also be physiologically stressful (Nabi et al., 2016). More research is necessary to investigate psychological mechanisms behind film preferences in individuals.

This is a rare study to access the association of film genre preference and the perceived stress in Chinese university students. The results of it have important implications to improve mental health by movies. Nonetheless, there are certain limitations to the current study that suggests assertions be accepted with some caution. First, the causal relationship between film genre preference and high perceived stress cannot be speculated based on the cross-sectional survey. Second, this survey was based on self-reporting by the participants, and recall bias may have been more pronounced in terms of their recollection of lifestyle. And perceived stress is associated with a variety of factors, such as academic achievement, lifestyle, while we did not take into account the examination results, the amount of cigarette and alcohol intake as the confounding factors in this study. Although, categorical variables were used whenever possible and the recall time was limited to the last month to minimize the recall bias of participating subjects. Further longitudinal and more detailed research is necessary for the future. Third, the film genres were not exhaustive, so more film genres choices should be provided. Furthermore, the use of self-reported data can potentially introduce recall bias, especially for variables based on participants’ long-term memory, which is also subject to dishonest responses or acquiescence bias whether they generalize to a broader population. Besides, although the PSS has shown high validity and reliability (Lu et al., 2017; Huang et al., 2020), a combination of physiological measurements may provide a better prediction of stress. Nevertheless, hopefully, the contributions of this research should not be overshadowed to the extant literature. Additionally, with the high pressure of mental health, the timely assessment of stress and other behavioral and mental illness using non-invasive tools like questionnaires (PSS) could timely help to assess the problems of the society.

## Conclusion

One year and a half after the COVID-19 pandemic, the perceived stress commonly existed among Chinese college students of this study. The personal preferences for certain film genres were significantly associated with perceived stress, even when controlling for confounding variables. A gender gap in different film genre preferences still exists. As for different film genre preferences, in particular, the preference for disaster movies, crime movies, and horror movies, is associated with higher perceived stress, it may turn out to be useful to pay more attention to an individual’s film viewing. And combining the findings of this study with physiological-psychological evidence, suspense movies might be used in mental health education interventions for college students to some extent. However, the mechanisms underlying this link remain largely unknown, and further researches including those in clinical settings seem warranted to investigate this question further. The teachers need to be concerned with the media usage history and preferences of their students and may advise students with high-level stress to avoid potentially harmful media content.

## Data Availability Statement

The original contributions presented in the study are included in the article/supplementary material, further inquiries can be directed to the corresponding author.

## Ethics Statement

This study was conducted by the Declaration of Helsinki, and it was approved by Ethics Review Committee (202106HS02). All the participants were vulnerable and provided in-formed consent before participating in the study based on the website. The patients/participants provided their written informed consent to participate in this study.

## Author Contributions

NQ conducted the statistical analyses of the data and wrote the all the manuscript and revision.

## Conflict of Interest

The author declares that the research was conducted in the absence of any commercial or financial relationships that could be construed as a potential conflict of interest.

## Publisher’s Note

All claims expressed in this article are solely those of the authors and do not necessarily represent those of their affiliated organizations, or those of the publisher, the editors and the reviewers. Any product that may be evaluated in this article, or claim that may be made by its manufacturer, is not guaranteed or endorsed by the publisher.
